# Identification and characteristics of a novel fosfomycin glutathione transferase, FosA12, from an MDR clinical isolate of *Proteus vulgaris*

**DOI:** 10.1128/aac.01138-25

**Published:** 2026-04-15

**Authors:** Wei Lu, Lihong Li, Xi Lin, Dongxin Liu, Keqing Zhang, Xinxin Zhou, Daojun Yu, Shenghai Wu

**Affiliations:** 1 Department of Laboratory Medicine, Hangzhou First People's Hospital Affiliated of Westlake University School of Medicine 74630, Hangzhou, China; 2 The 4th Clinical Medical College, Zhejiang Chinese Medical University 70571, Hangzhou, China; 3 Key Laboratory of Medical Genetics of Zhejiang Province, Key Laboratory of Laboratory Medicine, Ministry of Education, School of Laboratory Medicine and Life Sciences, Wenzhou Medical University 26453, Wenzhou, China; Entasis, Big Bay, Michigan, USA

**Keywords:** fosfomycin glutathione transferase, *Proteus vulgaris*, fosfomycin resistance, FosA12

## Abstract

This study identifies *fosA12*, a novel chromosomally encoded fosfomycin resistance gene in a multidrug-resistant *Proteus vulgaris* clinical isolate. Through whole-genome sequencing, *fosA12* was characterized, showing 64.23% homology with *Pseudomonas aeruginosa*’s *fosA*. FosA12, a glutathione S-transferase, confers high-level fosfomycin resistance (MIC 512 μg/mL) via five critical residues. These findings highlight *fosA12*’s role in resistance and the need for clinical surveillance.

## INTRODUCTION

The global rise in antibiotic resistance, particularly among multidrug-resistant pathogens, poses a significant public health challenge (1), complicating the development of new antibiotics (2, 3). Fosfomycin, an established antibiotic, is effective against various Gram-negative and Gram-positive bacteria, including MDR strains like methicillin-resistant *Staphylococcus aureus* and carbapenem-resistant *Enterobacteriaceae* (4, 5). However, fosfomycin resistance, driven by mechanisms, such as impaired drug transport, fosfomycin-modifying enzymes (e.g., FosA, FosB, FosX), efflux pumps, altered MurA binding, biofilms, and horizontal gene transfer, threatens its efficacy (6). In *Proteus vulgaris*, a Gram-negative pathogen linked to urinary tract infections, resistance arises from intrinsic chromosomal mutations and plasmid-encoded enzymes like FomA, FomB, and FosA, which inactivate fosfomycin (7). Notably, the FosA protein, a glutathione S-transferase, relies on manganese to confer resistance (8). Fosfomycin resistance is well-studied in *E. coli* and *K. pneumoniae* but less explored in *P. vulgaris* (9). This study identified a novel resistance gene, fosA12, in Proteus vulgaris through whole-genome sequencing and characterized the FosA12 enzyme *in vitro*. Results from these studies offer new insights into fosfomycin resistance in clinical settings.

*Proteus vulgaris* was cultured on blood agar and identified using Vitek-60 and 16S rRNA sequencing. *E. coli* BL21(DE3) and *DH5α* facilitated protein expression and cloning. Genomic DNA was sequenced, assembled with Canu v1.8, and analyzed using BWA and GATK (10). Genes were annotated via NCBI and DIAMOND (11). ResFinder and CARD detected resistance genes (12). Phylogenetic trees were built with MEGA X, visualized by iTOL (13). FosA12 motifs were analyzed using MEME Suite (14). Proksee and Easyfig created genome visualizations. FosA12 was PCR-amplified, mutated, cloned into pUCP20/pUCP24/pCold I, and expressed in *E. coli* BL21(DE3). FosA12 binding to glutathione and fosfomycin was assessed.

*Proteus vulgaris* G22 was isolated from urine culture of a clinical patient suffering from a urinary tract infection. For this strain, fosfomycin and cephalosporin were used first, and treatment was ineffective. After switching to imipenem and nitrofurantoin, the patient recovered. Subsequent minimum inhibitory concentration tests confirmed this strain’s sensitivity to meropenem, imipenem, trimethoprim-sulfamethoxazole, ciprofloxacin, and amikacin, but resistance to colistin and fosfomycin. A disk potentiation test with phosphonoformate, a FosA inhibitor, indicates that G22 contains potential fosfomycin resistance factors, as inhibition restored susceptibility (15, data not shown).

**Fig 1:**
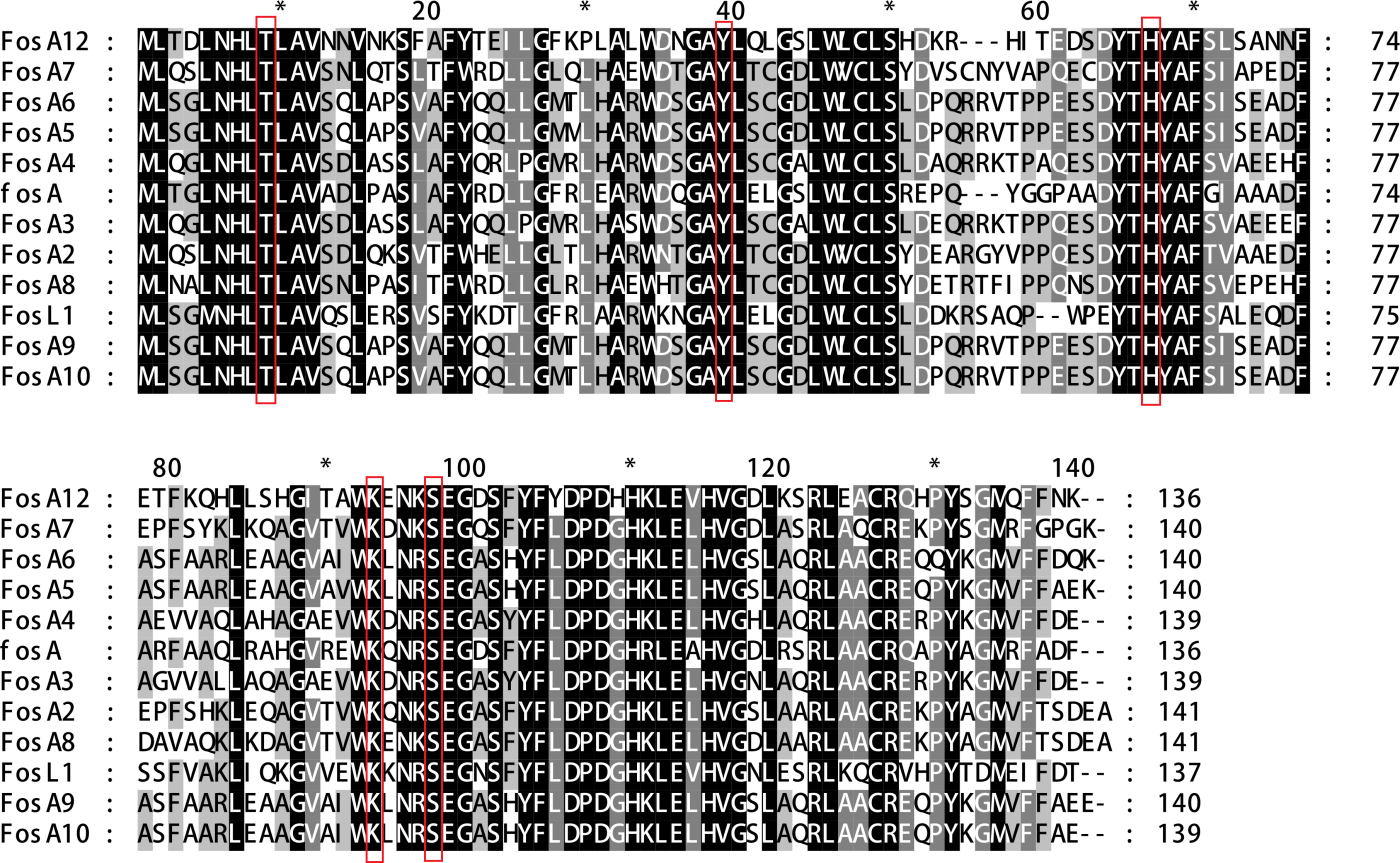
FosA multiple sequence alignment. Multiple sequence alignment of FosA proteins. Amino acid sequence alignment of FosA12 (PQ518868), FosA (AAA98399), FosA2 (ACC85616), FosA3 (AB522970), FosA4 (AB908992), FosA5 (AJE60855), FosA6 (NG051497), FosA7 (KKE03230), FosA8 (CP013990), FosA9 (PRJEB32329), FosA10 (MT074415), and FosL1 (QHR93773). The alignment was obtained using MEGA X. The black background indicates fully conserved residues; gray background indicates strongly similar residues. Numbers on the right correspond to the amino acid residues in each full-length protein.

*P. vulgaris* G22 strain’s 4.5 Mbp genome (GenBank: CP053371.1) has a 47.23% GC content, encoding 3,423 proteins, 6 rRNA, and 87 tRNA genes (Table S1). It shares 98.6% nucleotide identity with a related strain (CP079882.1). BLAST alignments against CARD and NCBI databases identified no known fosfomycin resistance genes, but three ORFs with lower identity were selected for resistance validation (Table S2). One of the genes has 58.82% identity with *fosC2* from *Aliidiomarina shirensis*, and the other has 54.17% identity with *fosC* from *Pseudomonas syringae.* Molecular cloning and drug sensitivity testing of overexpression strains based on published methods (16) revealed that neither gene had drug resistance. The last gene has a similarity of 64.23% with the *fosA* gene from *Pseudomonas aeruginosa*, showing high drug resistance activity against fosfomycin (17). BLAST results indicate that *fosA12* is present in 27 out of approximately 4,000 *Proteus* strains analyzed but absent in other *Enterobacterales*. The identity of *fosA12* with them ranged from 100% to 93%, indicating that the gene had low variability. Of these, 19 sequences are found in the *Proteus terrae* genome, while those in *Proteus appendicitidis* and *Proteus vulgaris* originate from human sources (Table S3). Notably, all these sequence results were predicted as vicinal oxygen chelate (VOC) superfamily of proteins, and the FosA enzymes belong to the VOC superfamily proteins, which encompasses a diverse group of metal-binding enzymes involved in processes like detoxification, antibiotic resistance, and other catalytic reactions involving vicinal oxygen chelates, but none have been subjected to in-depth research or validation (18). Finally, a novel fosfomycin resistance gene, *fosA12* (411 bp, 137 amino acids), was identified. Its share of highest amino acid sequence homology among known resistance genes is with the *fosA* gene from *Pseudomonas aeruginosa*, with a similarity of only 64.23%. It shows N-terminal conservation but C-terminal variation (Fig. 1). FosA12 raised fosfomycin MIC to 512 μg/mL when overexpressed in *E. coli* DH5α, with specific glutathione-dependent inactivation (Table 1; Table S4). Kinetic analysis revealed FosA12’s lower turnover rate (*k_cat_* = 63.3 s^–^¹) but higher substrate affinity (*K_m_* = 21 mM) compared with FosA5 (Table 1). The higher substrate affinity of FosA12 could enhance its efficacy in conferring antibiotic resistance, particularly in clinical settings where fosfomycin concentrations fluctuate. However, the lower catalytic efficiency (kcat/Km) of FosA12 compared with FosA5, if confirmed, might limit its overall performance under high substrate loads. Future studies should focus on elucidating the structural basis for FosA12’s enhanced affinity through X-ray crystallography or molecular dynamics simulations. These findings highlight the nuanced balance between substrate binding and catalysis in FosA enzymes, with potential implications for designing inhibitors targeting fosfomycin resistance mechanisms.

**TABLE 1:** Kinetic parameters of FosA12 and FosA5 in the assessment of the inactivation of fosfomycin^*a*^

Enzyme	MIC(μg/mL)	k_cat_ (s^−1^)	K_m_ (mM)	k_cat_/K_M_ (M^−1^ S^−1^)
FosA5	≥1,024	106.4 ± 3.2	32 ± 3	(3.3 ± 0.3) × 10^3^
FosA12	512	63.3 ± 4.6	21 ± 2	(3.0 ± 0.1) × 10^3^

^*a*^ The steady-state kinetic parameters of FosA12 and FosA5 for fosfomycin were determined in the presence of 30 mM glutathione. Kinetic parameters are reported as the means ± standard deviations from three or four independent biological replicates.

**Fig 2:**
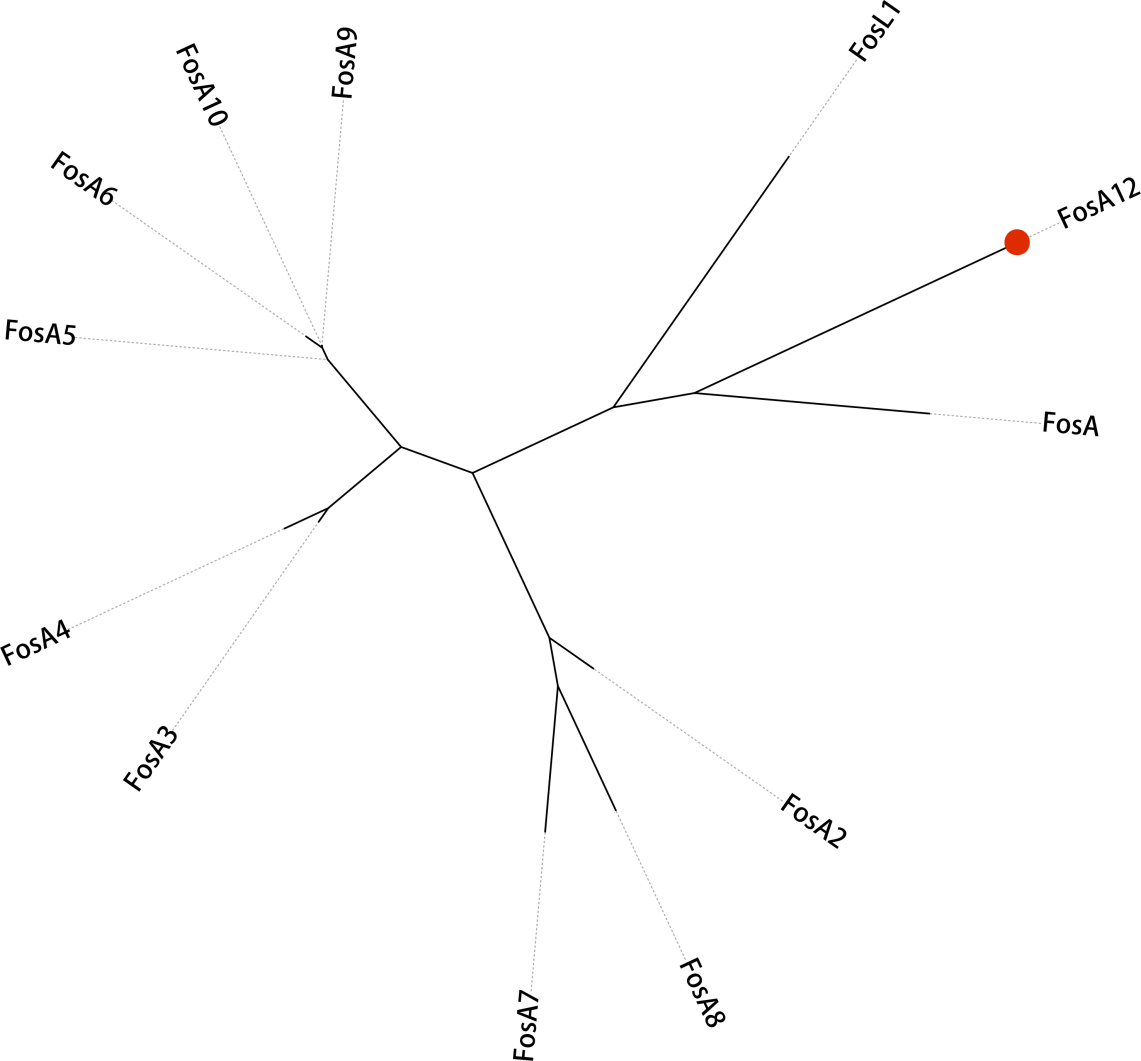
FosA phylogenetic tree. Phylogenetic analysis of FosA12 and all other 11 known FosA proteins. GenBank accession numbers are listed in Fig. 1. The bootstrap values are shown at the nodes of the tree. The scale bar represents a 10% amino acid sequence difference. FosA12 from this study is highlighted with a red filled circle.

Whole-genome sequencing revealed that the *fosA12* gene is chromosomally located in *Proteus vulgaris*, with no mobile genetic elements around its 40 kb flanking regions (Fig. S1). Comparative analysis showed >80% homology with other *Proteus vulgaris* strains, with *fosA12* (red in Fig. S1) flanked by functional and hypothetical protein-encoding genes. Previous studies have consistently reported that when a gene’s GC content deviates from the chromosomal average by more than 5%, it often indicates a horizontally transferred (HGT) origin (19). The distinct GC content difference was noted between *fosA12* and the chromosome. This phenomenon indicated FosA12 may have additional roles beyond fosfomycin modification, and its chromosomal integration raises concerns about potential spread to other species (20). Phylogenetic analysis (Fig. 2) positions FosA12 as a novel subclass, with >20% amino acid divergence from 11 FosA variants, distinct from FosA3/FosA9 clusters (21). Sequence alignment confirmed conservation of the LTLAV motif (residues 8–12) and Thr9 for Mn²^+^ coordination (22). His64 (58.3% conserved) and Ser94 (100% conserved) are critical, with H64A and S94A mutations reducing fosfomycin MIC by 128- and 256-fold, respectively (Table 2). Gly90, unique to FosA12, aids phosphate stabilization, with K90A causing a 256-fold MIC decrease. These residues (H64, S94, G90, T9, Y39) are essential for catalysis (Table 2). Despite ≤64% identity with FosA5/A10, FosA12’s conserved catalytic core and unique residues define its kinetic profile, emphasizing its role in fosfomycin resistance and the need for clinical surveillance (Fig. 1).

**TABLE 2:** The influence of site-directed mutations of key amino acid residues in *fosA12* on the resistance of fosfomycin^*a*^

Amino acid key residue	Mutation	Fosfomycin MIC (µg/mL)	Strain
Wild type: unmutated	None	512	DH5α/pUCP20/fosA12
Lysine (K90)	K90A	2	DH5α/pUCP20/fosA12(K90A)
Tyrosine (Y39)	Y39A	2	DH5α/pUCP20/fosA12(Y39A)
Serine (S94)	S94A	1	DH5α/pUCP20/fosA12(S94A)
Histidine (H64)	H64A	2	DH5/pUCP20/fosA12(H64A)
Threonine (T9)	T9A	2	DH5α/pUCP20/fosA12(T9A)

^*a*^ Relative susceptibility to fosfomycin was determined in E. coli DH5α overpressing either wildtype or mutant FosA12 enzymes.The catalytic core of FosA12 comprises five essential residues: Thr⁹ coordinates Mn²⁺ ions, His⁶⁴ stabilizes the metal-binding site, Ser⁹⁴ positions glutathione via hydrogen bonding, Tyr³⁹ facilitates hydrophobic substrate interactions, and Lys⁹⁰ electrostatically anchors fosfomycin's phosphate group. Alanine substitutions at each site reduced fosfomycin MIC by 64- to 128-fold, confirming their non-redundant roles in the enzymatic inactivation mechanism.

In summary, this study identifies *fosA12* as a novel chromosomally encoded fosfomycin resistance gene in *Proteus vulgaris*. FosA12, a glutathione S-transferase, relies on five key residues for its unique catalytic mechanism. It exhibits >10% amino acid divergence from known FosA variants, with no mobile genetic elements, confirming chromosomal integration. It’s fosfomycin-specific resistance. Phosphonoformate inhibition suggests potential for adjuvant therapy to manage resistance.

## Data Availability

Nucleotide sequence data reported are available in the third party annotation section of the DDBJ/ENA/GenBank databases under accession number PQ518868 (*fosA12*).

## SUPPLEMENTAL MATERIAL

Fig. S1 aac.01138-25-s0001.tif Genetic environment of *fosA12*.

Supplemental material aac.01138-25-s0002.docx Fig. S1 legend; Tables S1 to S3; data.

Table S4 aac.01138-25-s0003.xlsx Blast alignment results of *fosA12*.

ASM does not own the copyrights to Supplemental Material that may be linked to, or accessed through, an article. The authors have granted ASM a non-exclusive, world-wide license to publish the Supplemental Material files. Please contact the corresponding author directly for reuse.

## References

[1] Ferrara F, Castagna T, Pantolini B, Campanardi MC, Roperti M, Grotto A, Fattori M, Dal Maso L, Carrara F, Zambarbieri G, Zovi A, Capuozzo M, Langella R. 2024. The challenge of antimicrobial resistance (AMR): current status and future prospects. Naunyn Schmiedebergs Arch Pharmacol 397:9603–9615. doi:10.1007/s00210-024-03318-x 39052061

[2] Cook MA, Wright GD. 2022. The past, present, and future of antibiotics. Sci Transl Med 14:eabo7793. doi:10.1126/scitranslmed.abo7793 35947678

[3] Davies J. 2011. How to discover new antibiotics: harvesting the parvome. Curr Opin Chem Biol 15:5–10. doi:10.1016/j.cbpa.2010.11.001 21111668

[4] Bader MS, Loeb M, Leto D, Brooks AA. 2020. Treatment of urinary tract infections in the era of antimicrobial resistance and new antimicrobial agents. Postgrad Med 132:234–250. doi:10.1080/00325481.2019.1680052 31608743

[5] Pujol M, Miró J-M, Shaw E, Aguado J-M, San-Juan R, Puig-Asensio M, Pigrau C, Calbo E, Montejo M, Rodriguez-Álvarez R, et al.. 2021. Daptomycin plus fosfomycin versus daptomycin alone for methicillin-resistant Staphylococcus aureus bacteremia and endocarditis: a randomized clinical trial. Clin Infect Dis 72:1517–1525. doi:10.1093/cid/ciaa1081 32725216 PMC8096235

[6] Falagas ME, Athanasaki F, Voulgaris GL, Triarides NA, Vardakas KZ. 2019. Resistance to fosfomycin: mechanisms, frequency and clinical consequences. Int J Antimicrob Agents 53:22–28. doi:10.1016/j.ijantimicag.2018.09.013 30268576

[7] Gomez-Alferez A, Baquero F, Canton R, Loza E, Martinez-Beltran J. 1991. The incidence and beta-lactam resistance of Proteus vulgaris in hospital infections: the last decade. J Chemother 3:283–288. doi:10.1080/1120009x.1991.11739107 1809807

[8] Ito R, Mustapha MM, Tomich AD, Callaghan JD, McElheny CL, Mettus RT, Shanks RMQ, Sluis-Cremer N, Doi Y. 2017. Widespread fosfomycin resistance in gram-negative bacteria attributable to the chromosomal fosA gene. mBio 8:e00749-17. doi:10.1128/mBio.00749-17 28851843 PMC5574708

[9] Mattioni Marchetti V, Hrabak J, Bitar I. 2023. Fosfomycin resistance mechanisms in Enterobacterales: an increasing threat. Front Cell Infect Microbiol 13:1178547. doi:10.3389/fcimb.2023.1178547 37469601 PMC10352792

[10] Koren S, Walenz BP, Berlin K, Miller JR, Bergman NH, Phillippy AM. 2017. Canu: scalable and accurate long-read assembly via adaptive k-mer weighting and repeat separation. Genome Res 27:722–736. doi:10.1101/gr.215087.116 28298431 PMC5411767

[11] Hernández-Salmerón JE, Moreno-Hagelsieb G. 2020. Progress in quickly finding orthologs as reciprocal best hits: comparing blast, last, diamond and MMseqs2. BMC Genomics 21:741. doi:10.1186/s12864-020-07132-6 33099302 PMC7585182

[12] Florensa AF, Kaas RS, Clausen PTLC, Aytan-Aktug D, Aarestrup FM. 2022. ResFinder - an open online resource for identification of antimicrobial resistance genes in next-generation sequencing data and prediction of phenotypes from genotypes. Microb Genom 8:000748. doi:10.1099/mgen.0.000748 35072601 PMC8914360

[13] Letunic I, Bork P. 2021. Interactive tree of life (iTOL) v5: an online tool for phylogenetic tree display and annotation. Nucleic Acids Res 49:W293–W296. doi:10.1093/nar/gkab301 33885785 PMC8265157

[14] Bailey TL, Boden M, Buske FA, Frith M, Grant CE, Clementi L, Ren J, Li WW, Noble WS. 2009. MEME SUITE: tools for motif discovery and searching. Nucleic Acids Res 37:W202–W208. doi:10.1093/nar/gkp335 19458158 PMC2703892

[15] Ito R, Tomich AD, McElheny CL, Mettus RT, Sluis-Cremer N, Doi Y. 2017. Inhibition of fosfomycin resistance protein FosA by phosphonoformate (foscarnet) in multidrug-resistant gram-negative pathogens. Antimicrob Agents Chemother 61:12. doi:10.1128/AAC.01424-17 PMC5700359 28993329

[16] Yigit H, Queenan AM, Anderson GJ, Domenech-Sanchez A, Biddle JW, Steward CD, Alberti S, Bush K, Tenover FC. 2001. Novel carbapenem-hydrolyzing beta-lactamase, KPC-1, from a carbapenem-resistant strain of Klebsiella pneumoniae. Antimicrob Agents Chemother 45:1151–1161. doi:10.1128/AAC.45.4.1151-1161.2001 11257029 PMC90438

[17] Bharadwaj VG, Suvvari TK, Kandi V, P CR, Dharsandia MV. 2024. Molecular characterization of Pseudomonas aeruginosa clinical isolates through whole-genome sequencing: a comprehensive analysis of biotypes, sequence types, and antimicrobial and virulence genes. Cureus 16:e71118. doi:10.7759/cureus.71118 39525128 PMC11548977

[18] He P, Moran GR. 2011. Structural and mechanistic comparisons of the metal-binding members of the vicinal oxygen chelate (VOC) superfamily. J Inorg Biochem 105:1259–1272. doi:10.1016/j.jinorgbio.2011.06.006 21820381

[19] Douglas GM, Langille MGI. 2019. Current and promising approaches to identify horizontal gene transfer events in metagenomes. Genome Biol Evol 11:2750–2766. doi:10.1093/gbe/evz184 31504488 PMC6777429

[20] Allocati N, Favaloro B, Masulli M, Alexeyev MF, Di Ilio C. 2003. Proteus mirabilis glutathione S-transferase B1-1 is involved in protective mechanisms against oxidative and chemical stresses. Biochem J 373:305–311. doi:10.1042/BJ20030184 12667139 PMC1223472

[21] Hall RM, Schwarz S. 2016. Resistance gene naming and numbering: is it a new gene or not? J Antimicrob Chemother 71:569–571. doi:10.1093/jac/dkv351 26510717

[22] Klontz EH, Tomich AD, Günther S, Lemkul JA, Deredge D, Silverstein Z, Shaw JF, McElheny C, Doi Y, Wintrode PL, MacKerell AD Jr, Sluis-Cremer N, Sundberg EJ. 2017. Structure and dynamics of FosA-mediated fosfomycin resistance in Klebsiella pneumoniae and Escherichia coli. Antimicrob Agents Chemother 61:11. doi:10.1128/AAC.01572-17 PMC5655077 28874374

