# Supplementary figures and images for: Identification and characteristics of a novel fosfomycin glutathione transferase, FosA12, from an MDR clinical isolate of Proteus vulgaris
Source: Antimicrob Agents Chemother. 2026 Apr 15;70(5):e01138-25. doi: 10.1128/aac.01138-25 (PMC13148022; doi:10.1128/aac.01138-25)

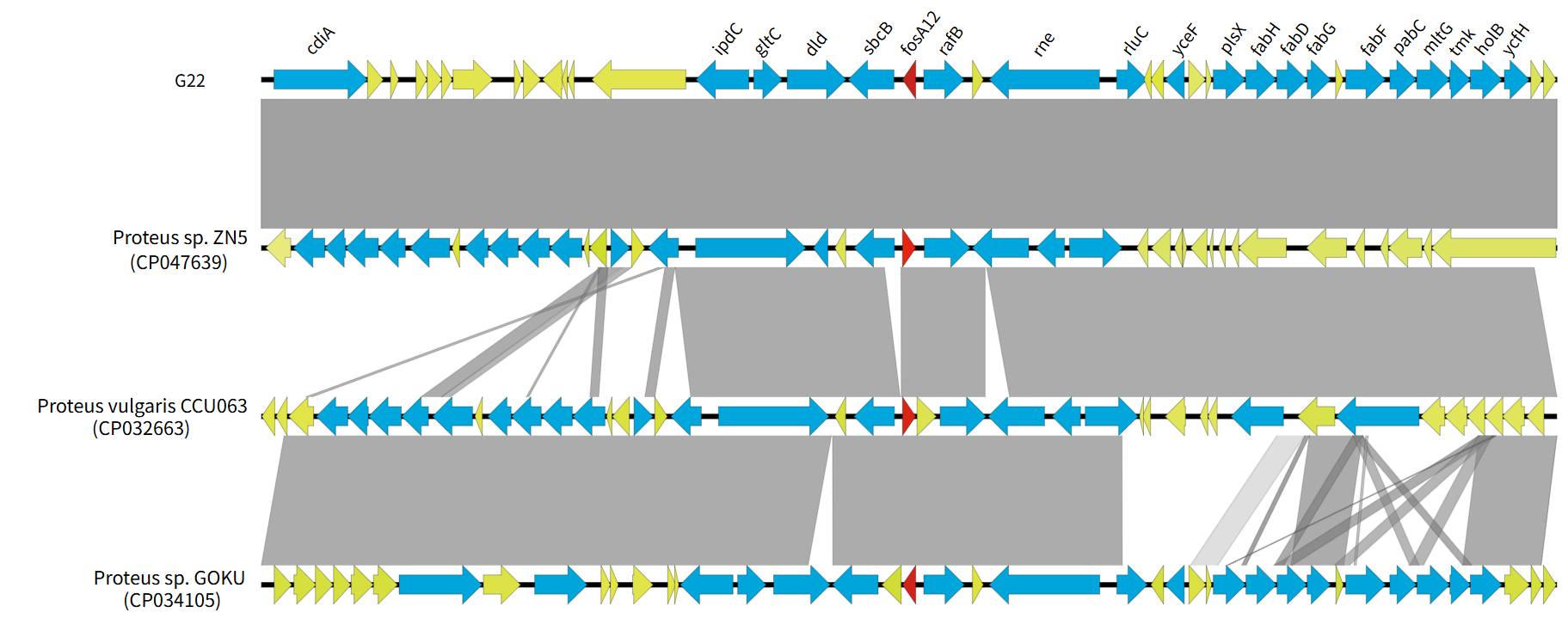

Supplement: Fig. S1 — Genetic environment of fosA12. [file aac.01138-25-s0001.tif]
