# Supplementary material for: Identification and characteristics of a novel fosfomycin glutathione transferase, FosA12, from an MDR clinical isolate of Proteus vulgaris
Source: Antimicrob Agents Chemother. 2026 Apr 15;70(5):e01138-25. doi: 10.1128/aac.01138-25 (PMC13148022; doi:10.1128/aac.01138-25)
Supplement: Supplemental material — Fig. S1 legend; Tables S1 to S3; data. [file aac.01138-25-s0002.docx]

**Figure S1.** **Genetic environment of** ***fosA12***

Schematic representation of the genetic environment of *fosA12* and comparison of the *fosA12-*carrying regions in genomes of *Proteus vulgaris* strains. ORFs are shown as arrows drawn to scale to indicate the direction of transcription. The *fosA12* gene is coloured in red and the other genes with known function are coloured in blue color. The ORFs encoding hypothetical proteins are coloured in yellow.

**Table S1. General features of the genome of *Proteus vulgaris* G22**

| **Characteristics** | **Chromosome** |
| --- | --- |
| Size (bp) | 4,515,465 |
| G+C % | 47.23 |
| Genes | 4,122 |
| ORFs | 4,122 |
| Known proteins | 3,423 |
| Hypothetical proteins | 692 |
| Average ORF length (bp) | 1045 |
| Average protein length (aa) | 343 |
| ncRNAs | 45 |
| ncRNA Regions | 28 |
| tmRNA | 1 |
| tRNAs | 87 |
| rRNAs | 16S*7; 23S*7; 5S*8 |

**Table S2: Putative novel resistance genes and antibiotic resistance phenotype**

| Antibiotics resistance pattern | Putative novel resistance genes | Length  (bp) | Identity  (%) | Observed resistance phenotypes*^b^* | GenPept accession no. of closest homologue |
| --- | --- | --- | --- | --- | --- |
| fosfomycin | *fosA-like* | 414 | 64.23 | fosfomycin | KP143090 [*fosA* Pseudomonas aeruginosa] |
|  | *fosC2-like* | 993 | 58.82 | — | WP_126805862.1 [*fosC2*  *Aliidiomarina shirensis*] |
|  | *fosC-like* | 1143 | 54.17 | — | AFM38990.1 [*fosC* *Pseudomonas syringae*] |

*a* —, no resistance to specific antibiotics was observed when expressed in *E. coli* DH5α

*b* Antibiotic resistance profiles of recombinant *E. coli* DH5α producing each putative resistance gene identified in this study.

**Tbable S3: Antimicrobial susceptibility test of *Proteus terrae* WX22 and recombinant strains harboring candidate resistance genes. (*mg/L*)**

| Strain | Fosfomycin*^a^* | Gentamicin | Streptomycin | Amikacin | Tobramycin | Ampicillin | Cefuroxime | Cefixime |
| --- | --- | --- | --- | --- | --- | --- | --- | --- |
| *E. coli* ATCC25922 | 1 | 0.5 | 4 | 1 | 0.5 | 4 | 2 | 0.06 |
| *Proteus terrae* WX22 | 1024 | 0.5 | 512 | 0.5 | 1 | 1024 | 1024 | 8 |
| *E. coli* DH5α/pUCP24*/* | 1 | 64 | 1 | 1 | 0.5 | 4 | 4 | 0.25 |
| *E. coli* DH5α/pUCP24*/fosA-like* | 512 | 64 | 1 | 1 | 0.5 | 4 | 4 | 0.5 |
| *E. coli* DH5α/pUCP24*/fosC2-like* | 1 | 64 | 1 | 0.5 | 0.5 | 4 | 4 | 0.5 |
| *E. coli* DH5α/pUCP24*/fosC-like* | 1 | 64 | 1 | 1 | 0.5 | 4 | 4 | 0.5 |

*a* Fosfomycin contain 25 ug/mL glucose 6-phosphate (G6P) according to CLSI standard.

**sequence data**

As the nucleotide sequence and derived amino acid sequence of the *fosA12* gene (GenBank accession no. PQ518868) is provided below for evaluation of the manuscript.

>Complete nucleotide sequence of the *fosA12 gene* Gene bank: PQ518868

ATGCTAACTGACTTAAACCACTTAACCCTCGCCGTCAATAATGTGAATAAAAGCTTTGCCTTTTATACTGAAATATTAGGTTTTAAGCCCCTTGCTTTATGGGATAACGGAGCTTACCTACAACTGGGTTCTTTATGGCTATGTTTATCTCACGATAAACGCCATATTACTGAAGACAGTGATTACACCCATTATGCTTTCAGTTTATCTGCAAATAATTTTGAAACCTTTAAACAGCATCTTTTATCCCATGGCATTACTGCATGGAAAGAAAATAAAAGTGAAGGTGATTCATTCTATTTTTACGATCCTGATCATCATAAACTTGAAGTCCATGTGGGTGATTTAAAAAGTCGCTTAGAAGCTTGTCGCCAGCATCCTTATTCAGGTATGCAATTTTTCAACAAATAA

>Complete amino acid sequence of FosA12

MLTDLNHLTLAVNNVNKSFAFYTEILGFKPLALWDNGAYLQLGSLWLCLSHDKRHITEDSDYTHYAFSLSANNFETFKQHLLSHGITAWKENKSEGDSFYFYDPDHHKLEVHVGDLKSRLEACRQHPYSGMQFFNK*
